# Navigated neuroendoscopy combined with intraoperative magnetic resonance cysternography for treatment of arachnoid cysts

**DOI:** 10.1007/s10143-019-01136-x

**Published:** 2019-07-16

**Authors:** Pawel Tabakow, Artur Weiser, Krzysztof Chmielak, Przemyslaw Blauciak, Joanna Bladowska, Marcin Czyz

**Affiliations:** 1 Department of Neurosurgery, Wroclaw Medical University, Borowska str. 213, 50-556 Wroclaw, Poland; 2 Department of General Radiology, Interventional Radiology and Neuroradiology, Wroclaw Medical University, Borowska str. 213, 50-556 Wroclaw, Poland; 3 Department of Neurosurgery, University Hospitals of Birmingham, Birmingham, UK

**Keywords:** Arachnoid cyst, Endoscopic cystostomy, Intraoperative magnetic resonance, Cysternography

## Abstract

Endoscopic cystocysternostomy or cystoventriculostomy is the treatment of choice in patients with symptomatic intracranial arachnoid cysts. There are no objective diagnostic tests for reliable intraoperative evaluation of the effectiveness of performed stomies. The aim of this prospective open-label study is to demonstrate for the first time the usefulness of intraoperative cysternography performed with the low-field 0.15-T magnetic resonance imager Polestar N20 during endoscopic cysternostomies. The study was performed in patients operated for middle fossa arachnoid cysts (*n* = 10), suprasellar cysts (*n* = 4), paraventricular or intraventricular cysts (*n* = 6), and a pineal cyst (*n* = 1). The operations were performed with use of a navigated neuroendoscope. Intraoperative magnetic resonance (iMR) cysternography was performed before and after the cystostomy. In each case, iMR cysternography was safe and could show clearly the cyst morphology and the effectiveness of performed endoscopic cystostomies. In six cases, iMR cysternography had a significant influence of the surgical decision (*p* = 0.027). The rate of inconsistency between the intraoperative observations and iMR imaging–based findings was 29%. A good contrast flow through the fenestrated cyst walls correlated with a good long-term clinical outcome (*ρ* = 0.54, *p* < 0.05) and good long-term radiological outcome (*ρ* = 0.72, *p* < 0.05). Intraoperative low-field MR cysternography is a safe and reliable method for assessment of the efficacy of performed endoscopic cystostomies and has significant influence on the surgical decision. It may be reliably used for prediction of the long-term clinical and radiological outcome.

## Introduction

Arachnoid cysts are congenital lesions that develop from splitting of the arachnoid membrane. Some of the cysts become symptomatic manifesting with headache, seizures, hydrocephalus (HCP), hemiparesis, visual loss, and hormonal and developmental disturbances. Presentation depends on the area they develop and the age of the patient [1]. Treatment modalities include endoscopic or microscopic cystocysternostomy or cystoventriclulostomy depending of the cyst localization or implantation of a shunt. A systematic review of the literature shows the superiority of cyst fenestration procedures over shunting. The latter is currently recommended only in cases of recurrent symptomatic cysts [2]. The choice of minimally invasive microsurgical versus endoscopic technique for cyst fenestration seems to depend mainly on the surgeons experience, and the results of treatment of the patients with both methods are similar for suprasellar, quadrigeminal, and posterior cranial fossa cysts, and slightly superior for Sylvian and cortical hemispheric cysts when treated microsurgically [3]. Neuroendoscopy is advocated by other authors as the treatment of choice of middle fossa cysts [4].

The application of image-guided navigation has increased the accuracy of targeting of the areas for cyst fenestration when normal anatomical landmarks are not identifiable, thus increasing the safety and effectiveness of the operations [5]. Yet there is no consensus regarding the size and number of cysternostomies to be performed [6, 7]. Together with the fact that intraoperative endoscopic exploration remains the main tool assessing the efficacy of cyst fenestration, it seems that the results from endoscopic cyst fenestrations are greatly dependent on the subjective surgeon intraoperative evaluation of the quality of performed fenestration. The introduction of real-time intraoperative magnetic resonance imaging (iMRI) during neuroendoscopy may be a useful solution for more reliable prediction of the success of cyst fenestration. The first attempts to combine neuroendoscopy with low-field iMRI were based on the usage of noncontrast T1- and T2-weighted imaging [8, 9]. We described the technique of intraoperative ventriculography combined with low-field iMRI imaging as a new method for more reliable assessment of the functionality of performed endoscopic third ventriculostomy (ETV) [10]. We showed that low-field iMRI ventriculography is a safe technique and may predict accurately the long-term outcomes of ETV. In this paper, we describe our clinical experience from the application of this new intraoperative diagnostic method in the treatment of patients with intracranial arachnoid cysts.

## Patients and methods

### Patients

Twenty-one patients with symptomatic arachnoid cysts were included in this prospective uncontrolled study. The patients were operated for Galassi type II or III middle fossa arachnoid cysts [11] (*n* = 10), suprasellar cysts (*n* = 4), paraventricular or intraventricular cysts (*n* = 6), and pineal cyst (*n* = 1). In all patients, the pathology was diagnosed based on high-field MRI studies. In each case, a written consent for an endoscopic procedure with use of iMR cysternography was obtained and a standardized questionnaire used routinely before MRI examinations was completed. In seven cases, the child’s parents gave the written consent for participation in the study. The study was approved by the Bioethics Committee of the Wroclaw Medical University in concordance with the Declaration of Helsinki.

### Surgical procedure

The patients were operated using a rigid 0° 6-mm neuroendoscope (Aesculap, Tuttlingen, Germany). It was used also as a navigation tool. The operations were performed in a shielded operating room adapted for iMR imaging. The 0.15-T MR imager Polestar N20 (Medtronic Navigation, Louisville, CO, USA) was used for intraoperative image acquisition. The detailed description of the technical considerations of use of the MR imager during the operations was described in our previous publications [8, 10, 12]. Briefly, the patients were placed in the supine position. In all except two infants, the head was fixed in a nonferromagnetic clamp and registered for navigation using preoperative high-field MRI scans. In case of the infants, a pinless head holder and a viscoelastic pad taped to the arms of the Polestar head holder were used to position the patient, as described previously [8]. Before the start of the operation, a positioning e-steady iMRI scan (24 s, 8 mm) was performed to determine the optimal position of the magnet during the surgery. Then, the scanner was draped and lowered below the operating table.

#### Middle fossa cysts

The trephination was performed above the cyst area according to the navigation plan. The cyst was punctured with an 0.8-mm-wide needle, and 5 ml from the cyst fluid was aspirated. In the next step, 5 ml of gadoteridol (0.5 mmol/ml, Prohance; Nycomed) diluted in Ringer’s solution (1:40) to a concentration of 0.012 mmol/ml was delivered to the cyst. A T1-weighted axial iMRI scan (three-dimensional gradient echo sequence, 4-mm slice thickness, 7-min acquisition time) was performed to show the degree of communication of the arachnoid cyst with the neighboring basal cisterns. In case of a noncommunicating cyst, a navigated endoscope was passed into the cyst. The cyst wall was first perforated using a sharp monopolar cannula, then enlarged with a blunt forcep and finally widened with a 3-French Fogarty balloon catheter. The surgical goal was to open all cyst membranes and to pass the 6-mm-wide endoscope trocar through the opening to the basal cisterns. The cysts were fenestrated mainly in the compartments between the internal carotid artery and oculomotor nerve or below the oculomotor nerve. By the time we were convinced that the fenestration has been adequately performed, a control iMR cysternography was performed. The contrast agent was delivered through a Cavafix catheter (B. Braun, Melsungen, Germany) passed through the working channel of the endoscope. The results from the MR cysternographic studies before and after the cyst fenestration were analyzed in the navigation system. Our criterion for an effective cysternostomy was the identification of contrast flow in the interpeduncular, perimesencephalic, and prepontine cisterns.

#### Suprasellar, pineal, and fourth ventricle cysts

The first step of the surgical procedure was a trephination, performed 2.5–3 cm lateral to the midline and at the level of the coronal suture. After the dura mater opening, the lateral ventricle was punctured with a brain needle for cerebrospinal fluid (CSF) aspiration and for contrast delivery. We chose the T1-weighted sagittal iMRI scans for optimal evaluation of cyst anatomy and contrast flow. In case of suprasellar cysts, our approach was first to fenestrate the cyst into the ventricle compartments and then to perform ETV. In case of a pineal cyst, we started first with ETV and then opened the cyst. For the latter maneuver, a second trephination was performed 4 cm anteriorly to the coronal trephination, to enable a safe introduction of the endoscope to the posterior part of the third ventricle. The patient with a fourth ventricle cyst underwent only cyst fenestration. After completion of the cystostomy in all cases except the case of fourth ventricle cyst, we assessed the efficacy of the surgery by observation of the oscillations of the third ventricle floor and also evaluated the area below the ventriculostomy for arachnoid membrane adhesions. The control iMRI scan was also sagittal T1-weighted, but this time, 5 ml of gadoteridol contrast was delivered either into the cyst (suprasellar and fourth ventricle cysts) or at the level of the foramen of Monro (pineal cyst). The criterion for an effective cyst fenestration, when ETV was part of the procedure, was the presence on sagittal scans of contrast agent passing into the inderpeduncular and prepontine cisterns and the presence of contrast on axial scans in the distally located arachnoid cisterns like the cerebellomedullary or Sylvian fissure cisterns. In the patient with a fourth ventricle cyst, we evaluated the quality of communication between the cyst and the ventricle.

#### Intraventricular and paraventricular cysts

The trephination was performed as close as possible to the cyst. After the cyst was punctured and 5 ml of CSF aspirated, 5 ml of diluted gadoteridol contrast was delivered into the cyst. A T1-weighted axial iMRI scan was performed to show the cyst topography. Then, the cystoventriculostomy was performed. A control axial iMRI scan was performed at the end of the procedure.

### Follow-up

The patients’ postoperative clinical state was evaluated in the period from the day of operation to discharge and at least 6 months after surgery. In patients in whom HCP was the main manifestation of the cyst, the treatment success was defined as an absence of clinical and radiological evidence of HCP in the long-term observation. Patients treated for middle fossa cysts were assessed for the presence of headache using the VAS score and for the presence of epilepsy or neurological impairment. The infants treated for an intraventricular cyst were examined monthly by a pediatric neurologist who evaluated their psychomotor development.

In each patient, a high-field 1.5-T (Signa HDXT, GE, USA) or 3-T MRI (Ingenia 3 T, Philips, Netherlands) study was performed in the long-term follow-up period. The presence of flow void phenomenon through the area of cyst fenestration on T2-weighted and heavily T2-weighted steady-state images (3D-FIESTA or 3D-DRIVE), the mean cyst volume, and the radiological signs of possible elevated intracranial pressure were assessed. The cyst volume was determined with the SmartBrush system (Brainlab, Germany) for object delineation and volume calculation. The main criteria for a good radiological result after the endoscopic procedure were the confirmation of cyst patency and the regression of radiological signs of cyst expansion like a decrease of the cyst volume, decrease of the midline shift, or the absence of HCP.

### Statistical analysis

All statistical analyses were performed with TIBCO Statistica™ (version 13.3; TIBCO Software Inc., Palo Alto, CA, USA) either by Spearman’s rank correlation test (using *ρ* coefficient), Wilcoxon signed-rank test, or chi-squared test. A value of *p* < 0.05 was considered statistically significant.

## Results

A summary of patient data, procedure, as well as clinical and radiological results is given in Table 1.

**Table 1:** Summary of patient’s file, procedure, and outcome

S. no.	Age (years)	Sex	Diagnosis	Symptoms	Preprocedure cysternography	Endoscopic procedure	Final postprocedure cysternography	Intraop. surgical decision*	Follow-up (months)	Complications**	Clinical result	Long-term MRI result
1	34	F	PVC	HA	NC	CV	GC	No	72	NR	I	I
2	50	F	MFC	HA	NC	CC	GC	No	66	NR	I	I
3	20	F	MFC	HA	NC	CC	GC	No	53	NR	I	I
4	26	M	MFC	HA, HCP	GC	Nd	Nd	Yes	61	NR	I	I
5	51	F	SSC	HA	NC	CV + ETV	GC	No	48	CSF leak	I	I
6	65	F	PVC	Memory, dysphasia	NC	CC	GC	No	60	CSF leak, meningitis	I	I
7	29	F	MFC	HA	NC	CC	GC	No	10	NR	I	I
8	36	F	MFC	HA	NC	CC	GC	No	36	NR	N	I
9	15	M	MFC	HA, left abducens nerve paresis	NC	CC	GC	No	48	NR	I	I
10	4	F	MFC	HA, dysphasia	NC	CC	GC	Yes	15	NR	I	I
11	15	M	MFC	HA, memory deficit	NC	CC	GC	No	26	NR	I	I
12	22	M	SSC	HCP, decrease of visual acuity	NC	CV + ETV	GC	No	20	Abducens nerve paresis	I	I
13	0.25	M	IVC	NR	NC	CV	GC	Yes	16	Pseudomeningocele	I	I
14	8	M	SSC	HCP, PI	NC	CV + ETV	GC	No	12	NR		I
15	31	M	IVC	HA, HCP, dysphasia, dysphagia, cranial nerve paresis	NC	CC	GC	Yes	50	NR	I	I
16	13	M	MFC	HA, LOC	NC	CC	GC	No	11	Paresis of oculomotor nerve	I	I
17	19	M	PC	HCP	NC	ETV + CV	GC	No	7	Memory deficit	I	I
18	21	F	SSC	HCP, visual acuity decrease	NC	CV + ETV	GC	Yes	6	NR	I	I
19	35	F	MFC	HA	NC	CC	GC	No	6	NR	N	I
20	66	F	PVC	Left hemiparesis	NC	CV	GC	No	6	NR	I	I
21	0.6	M	PVC	NR	NC	CV	GC	Yes	6	NR	W	W

*F* female, *M* male, *MFC* middle fossa cyst, *SSC* suprasellar cyst, *PVC* paraventricular cyst, *IVC* intraventricular cyst, *PC* pineal cyst, *HA* headache, *HCP* hydrocephalus, *PI* pituitary insufficiency, *LOC* loss of consciousness, *GC* good communication between the cyst and other arachnoid cisterns or ventricles, *NC* no communication between the cyst and other arachnoid cisterns or ventricles, *NR* none reported, *Nd* not done, *CC* cystocysternostomy, *CV* cystoventriculostomy, *ETV* endoscopic third ventriculostomy, *CSF* cerebrospinal fluid, *I* improvement of neurological state, *N* no change, *W* worsening of neurological state

*Yes means that the intraoperative MR cysternography influenced the surgeon decision to continue or to discontinue the endoscopic procedure

**All listed postoperative complications were reversible in a short period of time

### Safety

There were no adverse incidences noted during any of 21 operations of cystostomy. We did not observe any permanent neurological impairment in our patients. There was transient CSF leak through the wound in two patients (patients 5 and 6). One from these leaks resulted in subclinical meningitis (asymptomatic CSF pleocytosis) that was successfully treated pharmacologically. There was one case of transient paresis of the oculomotor nerve and one case of abducens nerve paresis that lasted several hours (patients 16 and 12), one case of transient memory deficit (patient 17), and one case of pseudomeningocele in a 3-month-old infant with an intraventricular cyst (patient 13). He underwent recysternoventriculostomy and surgical closure of the pseudomeningocele. We did not observe any adverse events directly related to the usage of the iMR cysternography.

### Diagnostic accuracy of iMR cysternography

**Fig. 1:**
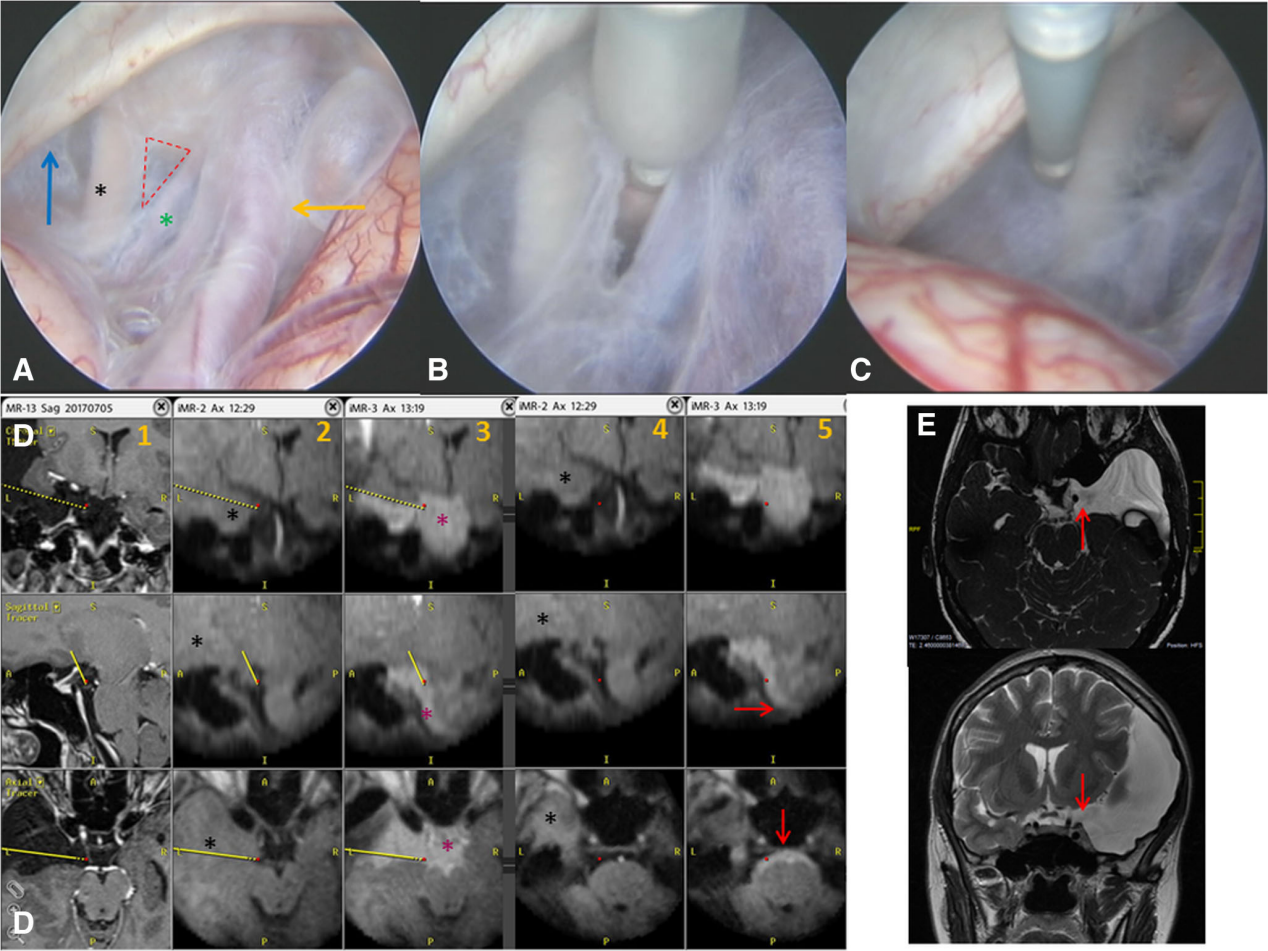
Images showing the endoscopic cystocysternostomy performed in a 35-year-old woman (patient 19) suffering from a large middle fossa arachnoid cyst. **a** Endoscopic view from the inside of the cyst. The red dotted lines are pointing the triangular area of planned fenestration, between the left oculomotor nerve (black asterisk) and the posterior communicating artery (green asterisk). The yellow arrow is showing the internal carotid artery while the blue one the anterior petroclinoidal fold. **b** The cyst fenestration is enlarged with a 3F Fogarthy catheter. **c** The catheter is exploring the area between the oculomotor nerve and the petroclinoidal fold for a possible second cyst fenestration. Yet based on the positive results from iMR cysternography that showed an effective contrast flow through the first fenestration (D), we resigned from performing a second one. **d** Radiological view of the arachnoid cyst before its fenestration (column 1—high-field 1.5-T images, columns 2 and 4—low-field intraoperative 0.15-T images after contrast administration into the cyst; the area containing the diluted gadolinium contrast is pointed with a black asterisk) and after the cystocysternostomy (columns 3 and 5). The yellow line is showing the surgical plan for cyst fenestration. Prefenestration intraoperative MR cysternography demonstrated no contrast flow from the cyst to the neighboring cysterns, while the postfenestration cysternography confirmed clearly an efficient contrast flow not only to the arachnoid cysts localized close to the fenestration point (column 3, pink asterisk) but also in more distally located cysts (column 5, red arrow). **e** Postoperative axial heavily T2-weighted steady-state image (3D-DRIVE), and T2-weighted coronal 3-T MRI scans performed 6 months after surgery. Red arrows point the side of cyst fenestration with visible flow void effect. We observed additionally a decrease of the cyst volume from 127.3 to 115.3 cm^3^ and a decrease of the midline shift from 4.5 to 3 mm

**Fig. 2:**
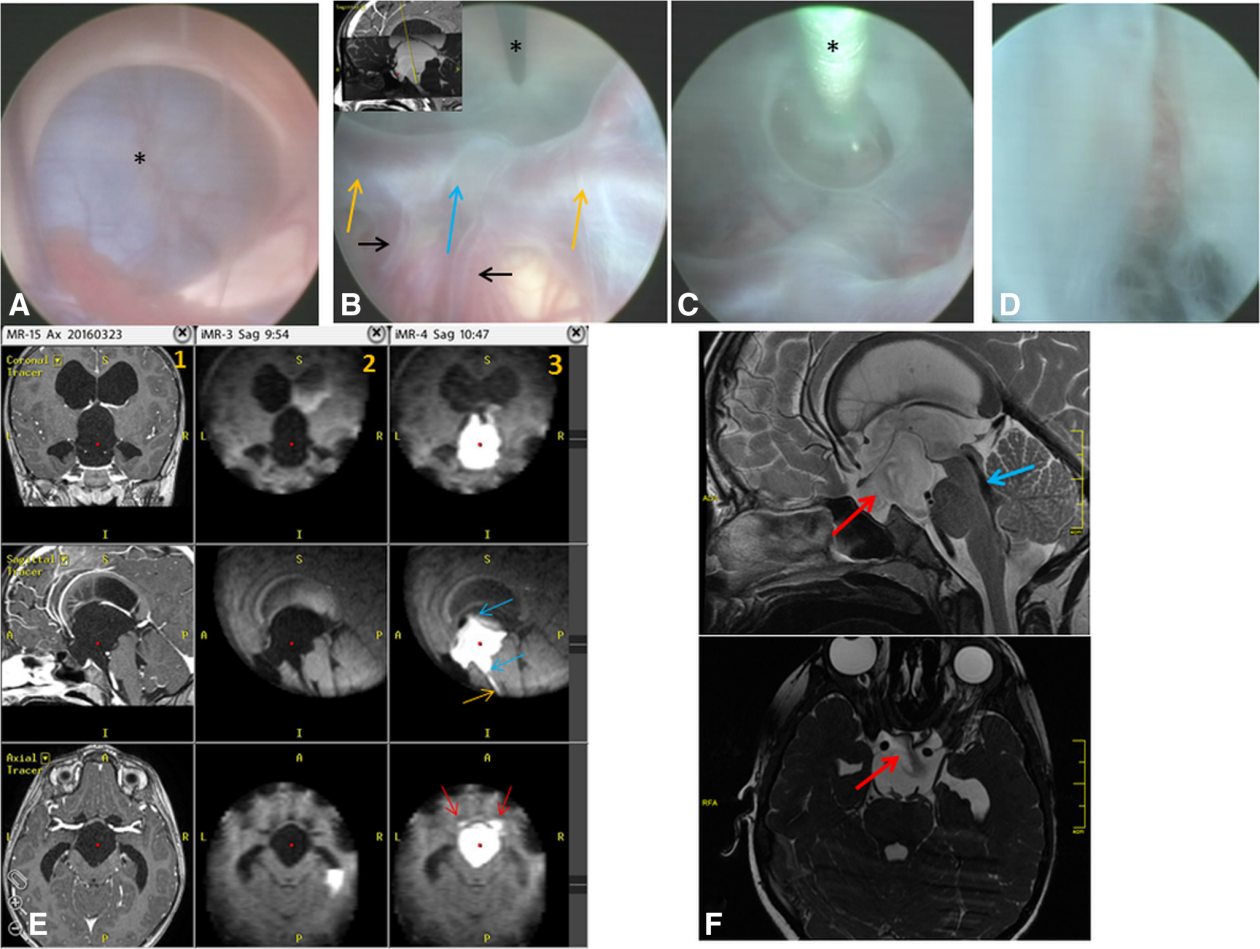
Images demonstrating the endoscopic procedure performed in a 8-year-old boy (patient 14) suffering from HCP and pituitary insufficiency due to a large suprasellar cyst. **a** Endoscopic view of the right lateral ventricle occluded by an arachnoid cyst bulging through the foramen of Monroe (asterisk). **b** View of the cyst floor after the performance of endoscopic perforation of the upper cyst wall at the level of foramen of Monro. MRI T2-weighted scan in a sagittal plane, presented in the left upper corner, is showing the plan for bilateral cyst perforation. Note how deep the cyst penetrates the space between the brain stem and clivus. The basilar artery tip (blue arrow), together with both posterior arteries (yellow arrows) and their deep branches (black arrows), are located relatively high to the site planned for cystostomy pointed by a monopolar probe (asterisk). **c** The 3F Fogarthy balloon catheter (asterisk) is enlarging the fenestration in the narrow space between the trunk of the basilar artery and clivus. **d** Final view of the fenestration. **e** MRI scans of the arachnoid cyst before its fenestration (column 1—high-field 1.5-T images; column 2—low-field intraoperative 0.15-T images after contrast administration into the right lateral ventricle) and after the cystoventriculostomy (column 3). The cyst initially was totally separated from the arachnoid and ventricular system blocking the cerebrospinal fluid flow through the third ventricle towards the aqueduct (columns 1 and 2). After the bilateral cyst fenestration (column 3, blue arrows), the contrast traverses the cyst and flows to the arachnoid space of the prepontine area (yellow arrow). The cyst has shrinked in comparison with its preoperative shape. Red arrows show the distal contrast flow reaching the Sylvian fissure arachnoid space. **f** Postoperative high-field sagittal T2-weighted and axial heavily T2-weighted steady-state (3D-DRIVE) MRI scans performed 1 year after the surgery. Note the evident shrinkage of the cyst volume enabling a free flow of CSF through the third ventricle. Red arrows show the flow void phenomenon through the fenestrated cyst while the blue one the flow void through the Sylvian aqueduct

**Fig. 3:**
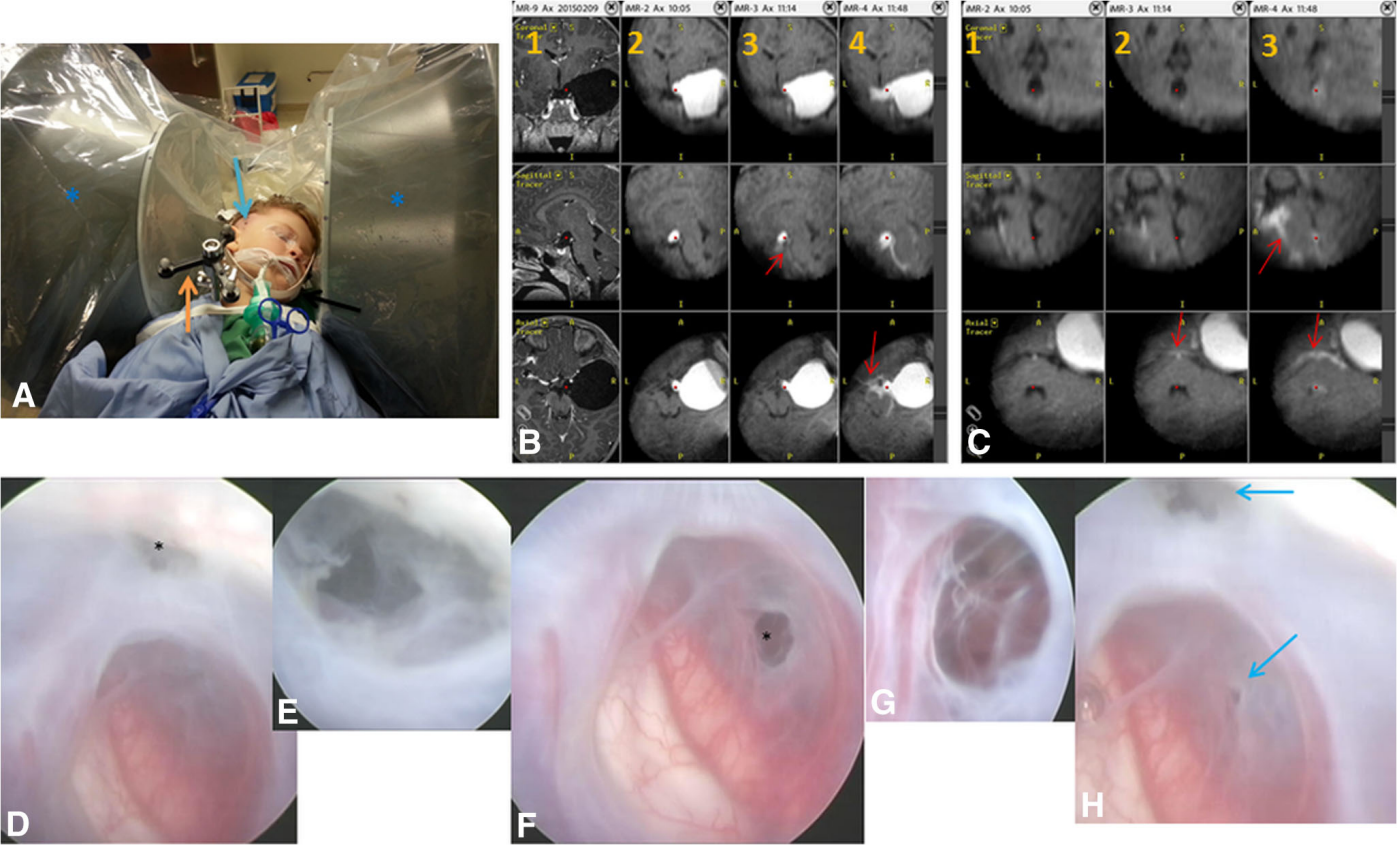
A 4-year-old girl with an arachnoid cyst of the right middle fossa (patient 10). **a** Photo showing the patient positioning. The head was fixed in a nonferomagnetic clamp. A receive coil was placed on the head around the operative field and connected to the low-field iMR imager (black arrow). Orange arrow is pointing the passive navigation reference frame. Blue arrow is showing on the skin the planned surgical entry point. The intraoperative MR scanner (blue asterisk) is placed in a position ready for the performance of the first positioning scan. **b** Column 1—preoperative high-field T1-weighted contrast enhanced scan showing the expansive character of the cyst. Columns 2, 3, and 4 show the result from low-field iMRI imaging. The contrast delivery into the cyst shows no patency of the arachnoid cyst (column 2). After initial cyst fenestration, there is only some insufficient contrast flow through the cyst (column 3, red arrow). The performance of a second larger fenestration resulted in evident contrast flow from the cyst to the neighboring arachnoid cisterns (column 4, red arrow). **c** Further intraoperative images comparing the prefenestration cyst appearance (column 1), the appearance after the first fenestration with minimal contrast flow (column 2, red arrow), and the improved contrast flow into more distally located arachnoid cisterns after the conduction of the second fenestration (column 3, red arrow). **d** Intraoperative endoscopic view of the inner part of the cyst. The asterisk points the initially performed fenestration, in proximity to the petroclinoidal fold. **e** Enlargement of the area of fenestration shown in **d**. **f** View of the second fenestration (asterisk) conducted after obtaining of the result from iMRI cysternography that showed insufficient contrast flow through the first stoma. The second stoma was performed between the posterior communicating and choroidal artery. **g** Enlargement of the area of fenestration shown in **f**. **h** View of both fenestrations (blue arrows)

We found iMR cysternography as a very useful tool that could demonstrate both the cyst morphology as well as the degree of cyst patency before and after its marsupialization. Figure 1 shows an exemplary case of cystocysternostomy, while Fig. 2 demonstrates the case of cysternoventriculostomy. In both examples, cysternography confirmed the patency of the endoscopic fenestration. Apart from confirmation of the surgical goal, the results from iMR cysternography influenced the surgeon’s intraoperative decision in six cases. The rate of change of the surgical goals based on iMR findings was statistically significant (*p* = 0.027, Table 1). In four cases (patients 10, 13, 15, and 21), the first postfenestration iMR cysternography did not show efficient flow of the contrast through the stoma, which resulted in continuation of the surgical procedure until the desired radiological effect was obtained (Fig. 3). In two cases, iMR cysternography showed either that the arachnoid cyst had been patent before surgery (patient 4), which could not be detected on preoperative MRI scans, or that there is no need to enlarge the performed stoma or to perform a second one (patient 18).

### Clinical outcome

The patency of the cytostomy proven by the iMR cysternography positively correlated with the long-term clinical outcome (*ρ* = 0.54, *p* < 0.05). In all patients, except three, in whom the cysternography showed good contrast across the performed cyst fenestration, a clinical improvement was observed postoperatively. In patients 8 and 19, treated for a middle fossa cyst, the headache persisted postoperatively, although there was a decrease of the cyst size and the midline shift on the follow-up imaging.

In patient 21, who was treated for two paraventricular cysts, a right-sided hemiparesis was noted 6 months postoperatively. MRI study showed an increase of the midline shift and cyst volume. The patient underwent an endoscopic revision cystostomy, but because of the occurrence of postoperative subdural hygromas, he underwent finally an implantation of a subdural-peritoneal shunt. He presented good recovery to his motor power and overall performance.

### Radiological findings

**Fig. 4:**
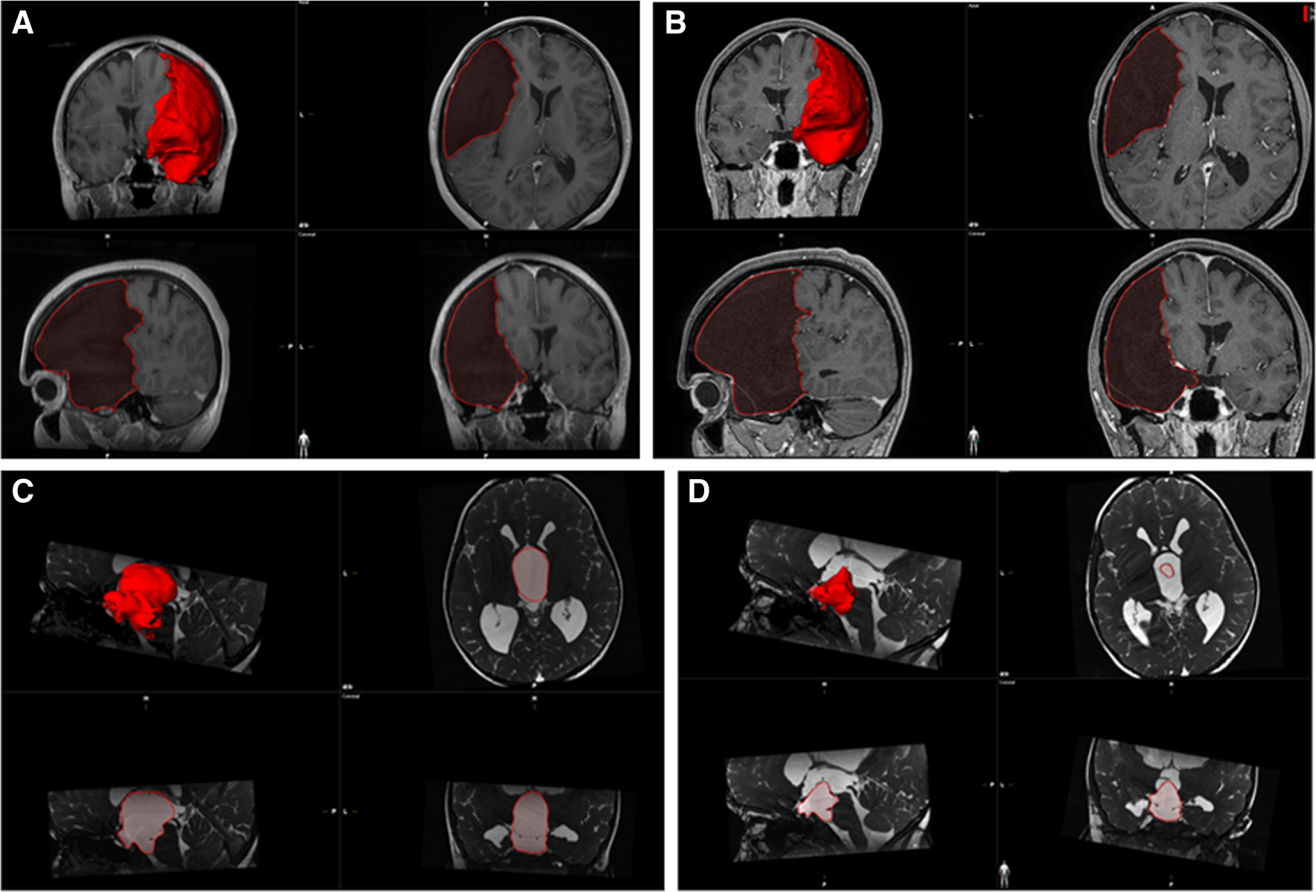
Images showing the method for three-dimensional cyst visualization and calculation of its volume with application of the the SmartBrush software (Brainlab, Germany). The 3D T1-weighted contrast enhanced images **a** (preoperative) and **b** (postoperative) concern patient 8. In this case, there was a small but detectable decrease of the cyst volume postoperatively from 269.9 to 256.9 cm^3^ as well as a minimal decrease of the midline shift from 6 to 5.5 mm. This patient had persistent headache in the long-term 3-year follow-up. The T2-weighted steady-state (3D-DRIVE) images **c** (preoperative) and **d** (postoperative) demonstrate the radiological results from treatment of patent 14. Postoperatively, there was a substantial decrease of the cyst volume (from 43.4 to 18.2 cm^3^) as well as evident flow void through the cyst 1 year after the surgery. There was no midline shift both before and after surgery. The patient improved neurologically, presenting no symptoms of HCP and minimal somatotropine pituitary insufficiency, controlled with growth hormone substitution

A summary of the preoperative and postoperative radiological findings is presented in Table 2. In 19 cases (90.4%), we observed a significant decrease of the cyst volume postoperatively (*p* = 0.005, Fig. 4). In 15 cases (71.4%), this decrease of the cyst volume was accompanied by a decrease of the midline shift (*p* = 0.004). In 13 cases (61.9%), a flow void phenomenon of CSF was detected that confirmed the cyst patency (*p* = 0.001). There was a high positive correlation between final postfenestration iMR cysternography result and the long-term MRI result (*ρ* = 0.72, *p* < 0.05).

**Table 2:** Comparison of the parameters of cyst volume, shift of the midline brain structures, and the presence of cyst patency before and after the endoscopic fenestration procedure

S. no.	Diagnosis	Preoperative cyst volume (cm^3^)	Preoperative midline shift (mm)	Preoperative flow void in the cyst (yes = 1, no = 0)	Postoperative cyst volume (cm^3^)	Postoperative midline shift (mm)	Postoperative flow void in the cyst (yes = 1, no = 0)
1	PVC	233.5	5	0	200.7	4	1
2	MFC	234.6	7.5	0	204.1	3	0
3	MFC	86.2	5.5	0	42.2	3.5	0
4	MFC	46.9	5	0	46.7	3.5	0
5	SSC	266.8	0	0	232.2	0	1
6	PVC	147.5	13.5	0	69.8	4	1
7	MFC	327.1	5	0	314	3.5	1
8	MFC	269.9	6	0	256.9	5.5	1
9	MFC	220.1	9	0	120.8	5	0
10	MFC	76	1.5	0	10.2	− 3**	1
11	MFC	118.2	4	0	90.6	1	1
12	SSC	15.1	1.5	0	8.9	1.5	1
13	IVC	294.3	16.6	0	340.3	11.8	0
14	SSC	43.4	0	0	18.2	0	1
15	IVC	13.9	0	0	2.3	0	1
16	MFC	172.1	7	0	70.3	1	0
17	PC	1.96	0	0	0.3	0	1
18	SSC	13.1	0	0	8.47	0	1
19	MFC	127.3	4.5	0	115.3	3	1
20	PVC	108.6	9	0	18.5	2.5	0
21	PVC	272.5	3	0	620.3	7.5	0/1*

The data in the gray columns concern the postoperative radiological findings performed at least 6 months after the surgery

*MFC* middle fossa cyst, *SSC* suprasellar cyst, *PVC* paraventricular cyst, *IVC* intraventricular cyst, *PC* pineal cyst

*There was flow void between the two single paraventricular arachnoid cysts but no flow void into the ventricle

**The midline shift was ipsilateral as a result of substantial decrease of the cyst volume

## Discussion

In this study, we demonstrated that endoscopic fenestration of symptomatic arachnoid cysts is a safe and effective procedure, obtaining high rates of good clinical (85.7%) and radiological outcome (95%). In patients with suprasellar and pineal cysts, 100% of good clinical and radiological long-term results were observed, while the success rate in patients with paraventricular or intraventricular cysts was 83.3%, and in patients with middle fossa cysts, it was 80%. These results are in accordance with observations from other studies indicating patients with symptomatic suprasellar, quadrigeminal, and paraventricular and intraventricular cysts as most beneficial candidates for endoscopic treatment and patients with middle fossa cysts as less predictable responders to endoscopic fenestration [3, 7, 13]. A confirmation of this thesis is our observation that both patients with a middle fossa cyst (patients 8 and 19) had been complaining of ongoing headaches several months postoperatively even though the follow-up MRI studies showed a decrease of the cyst size and the midline shift. Of note, one of these patients had presented with ongoing social issues that might have influenced the overall outcome.

The high rate of good clinical and radiological improvement observed in our patients was most likely influenced by the usage of the technique of low-field iMR cysternography. The four cases where there was a clear need to continue the fenestration procedure to obtain efficient contrast flow through the stoma as well as the two cases where such procedure was not necessary at all (because the cysts were patent) or where there was no need to continue the surgery prove the potential usefulness of iMR cysternography. Similar observations in our cohort of patients who had undergone ETV with application of low-field iMR ventriculography seem to confirm this thesis even further [10]. In three patients of the group of 11 who underwent ETV, intraoperative assessment of the patency of the ventriculostomy was inconsistent with the results of iMR ventriculography. The rate of inconsistency between the intraoperative surgical observations and iMR imaging–based findings was very similar in both studies, being 27% [10] and 29%, respectively. Apart from the similarity in terms of clinical usefulness, both studies confirmed the safety of iMR ventriculography and cysternography. All adverse postoperative events were mild and transient and were attributed to the fact of usage of the neuroendoscopic technique rather than the fact of application of diluted paramagnetic contrast.

Intraoperative MR cysternography could predict the long-term clinical outcome (*ρ* = 0.54, *p* < 0.05) and more precisely the long-term radiological outcome (*ρ* = 0.72, *p* < 0.05). Besides, it could increase the objectivity of the surgical decisions whether to carry on or not the further exploration in the region of stoma. According to our knowledge, there is no reliable method based on preoperative clinical and demographic factors or intraoperative observations that could predict the success rate of endoscopic cystocysternostomy or cystoventriculostomy. Such methods have been described only for the prediction of the ETV success rate and include the ETV success score [14, 15] and the grading score developed by Greenfield et al. [16].

We did not use the iMRI scans for readjustment of our navigation plan. Draining of CSF for the performance of cysternography as well as for endoscopic cyst fenestration did not cause significant shift of the brain structures, especially in areas of the cysts where the targets for fenestration were established. This may be explained by the fact that the technique of skull opening (trephination), the cyst topography (in most cases localized close to the skull base), and the fact of constant cyst irrigation with the endoscope) may have decreased the probability of brain shift. Thus, we could reliably use navigation based on preoperative high-field MRI scans and applied iMR cysternography mainly for evaluation of cyst morphology and the effectiveness of performed endoscopic cystostomies. Yet, in our previous work, we demonstrated that in case of lack of preoperative navigation high-field MRI images, low-field iMRI can be effectively used for intraoperative navigated endoscopy [8].

Our study reproduces the results of others treating patients with suprasellar and quadrigeminal cysts. The lower success rate of endoscopic treatment of middle fossa cyst described in the literature and confirmed also in our study can be explained by the relatively unspecific clinical presentation as well as by the anatomical restraints limiting the possibility of obtaining an effective cystocysternostomy. The main operative approach is to perform as many as possible fenestrations and as large as possible [17]. Certainly, this goal cannot be achieved in every single case [17]. Attempts to increase the number and size of the performed stomies may increase the risk of complications given the topography of the area of interest. Additionally, endoscopic fenestrations are in general smaller than the microsurgical ones because of the coaxial working trajectory of the endoscope along the working channel [17]. Introduction of the low-field iMR cystermography may enable a reliable prediction of the long-term outcomes and eliminate the need for unnecessary surgical manipulations.

The two cases of infants with intraventricular cysts deserve a special attention. Unfortunately, in both cases, a revision surgery was required. In patient 13, a pseudomeningocoele has developed postoperatively. It was most likely caused by the fact that trephination and dural opening in a cruciate fashion was used which did not allow us to perform a watertight dural closure. The pseudomeningocoele might have caused a decrease of the pressure gradient at the level of the stoma and its subsequent obliteration. A revision endoscopic fenestration through a key hole craniotomy was performed, followed by a watertight dural reconstruction using a dural substitute. The procedure was successful, and good long-term clinical outcome was noted. Patient 21 was operated for two symptomatic paraventricular arachnoid cysts, which were surgically connected with the third ventricle. The patency of stoma was proven by the iMR cysternography. Six months postoperatively, the child developed a right-sided hemiparesis. The MRI revealed a marked increase of the midline shift and cyst volume. During the revision endoscopic surgery, iMR cysternography confirmed lack of communication between the cysts and the ventricular system. A new fenestration was performed directly into the left lateral ventricle, and its patency was again confirmed with iMR cysternography. Yet, an expansive subdural hygroma developed following the surgery, requiring the insertion of a subduro-peritoneal shunt. The reason for the obliteration of the stoma remains unclear to us. In larger series of children treated for intracranial arachnoid cysts, the failure of endoscopic fenestration in infants was 44% and was significantly higher (*p* < 0.001) than in other age groups (4%) [18]. This group also showed the highest rate of postoperative complications requiring additional surgery (71.4%). Interestingly, the success rate of cysternostomies in infants is comparable with the efficacy of ETV and is unsatisfactory [14, 15, 18]. This observation may be partially explained by the underdeveloped mechanisms of the CSF absorption [19] and by the intense self-repair ability of the glial and connective tissue leading to spontaneous obliteration of the stoma with time [20, 21].

There are few limitations to our study. Our relatively small cohort of patients was heterogeneous with regard to the cyst type and age of patients treated. That makes the statistical analysis underpowered and difficult to be directly transferred into the clinical routine. This fact also may explain the observation that while iMR cysternography significantly influenced the surgical decision in six of the described cases, it did not improve the overall outcome. Besides, it has to be noted that in two from these six cases, the change of surgical decision was not to continue the surgery, because the cysternographic study showed that the stoma was preexisting (patient 4) or good enough (patient 18). Thus, iMR cysternography improved in these cases rather the safety but not the outcome of our procedure by preventing unnecessary surgical manipulations. Another limitation of our study is that it presents a single-center experience with no control group of patients treated in a “classic” way (endoscopic marsupialization without iMRI). We compared our results with those available in the literature, and the variability of cases represents in our opinion quite well real-life clinical scenarios. In order to provide higher level of evidence, analysis of larger and more homogeneous groups of patients is warranted. At present, according to our knowledge, we introduce the very first series of patients treated with the method that may contribute to improved outcomes and safety.

## Conclusion

Endoscopic intracranial arachnoid cyst fenestration with use of low-field iMR cysternography was safe and effective for all types of treated cysts. Intraoperative cysternography essentially influenced the surgical decision and could predict adequately the long-term clinical and radiological outcome. The low-field iMR cysternography may be a very helpful adjunct in determination of the surgical goals and limiting the endoscopic manipulations to absolute minimum. A prospective multicentre study evaluating the usefulness of low-field iMR imaging during endoscopic cysternostomy conducted in a homogenic group of patients is advocated.
